# Supplementary material for: Cancer Cells Shuttle Extracellular Vesicles Containing Oncogenic Mutant p53 Proteins to the Tumor Microenvironment
Source: Cancers (Basel). 2021 Jun 15;13(12):2985. doi: 10.3390/cancers13122985 (PMC8232660; doi:10.3390/cancers13122985)

Figure S1. The whole Western Blot figure of Figure 1A

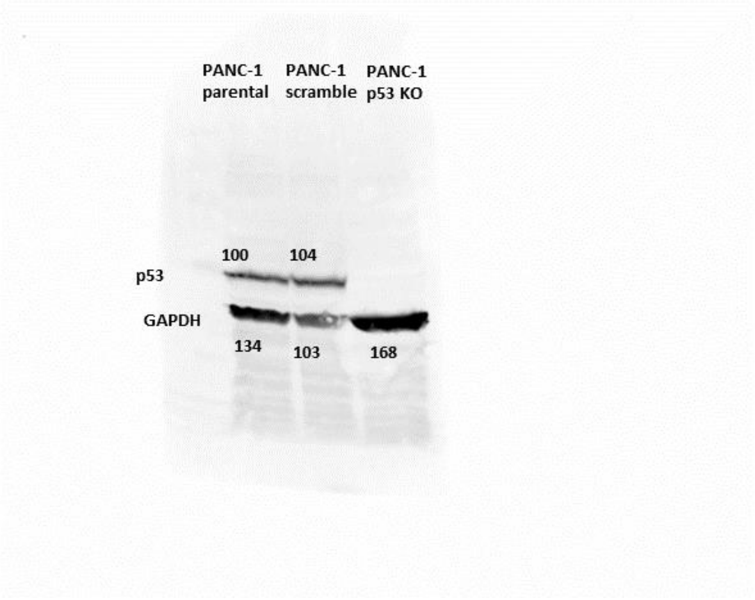

Figure S2. The whole Western Blot figure of Figure 2A

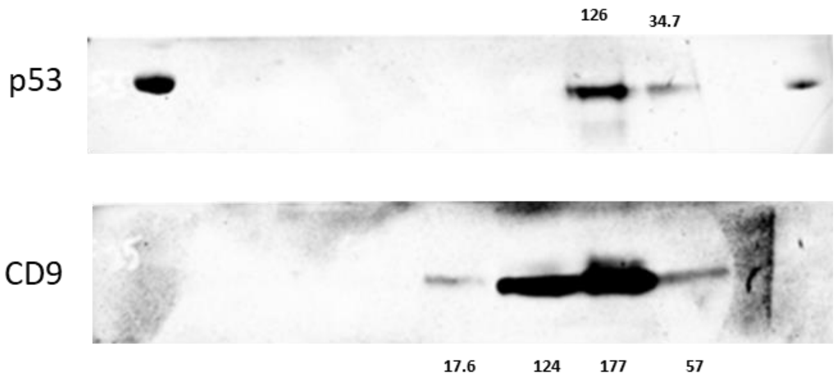

Figure S3. The whole Western Blot figure of Figure 2B

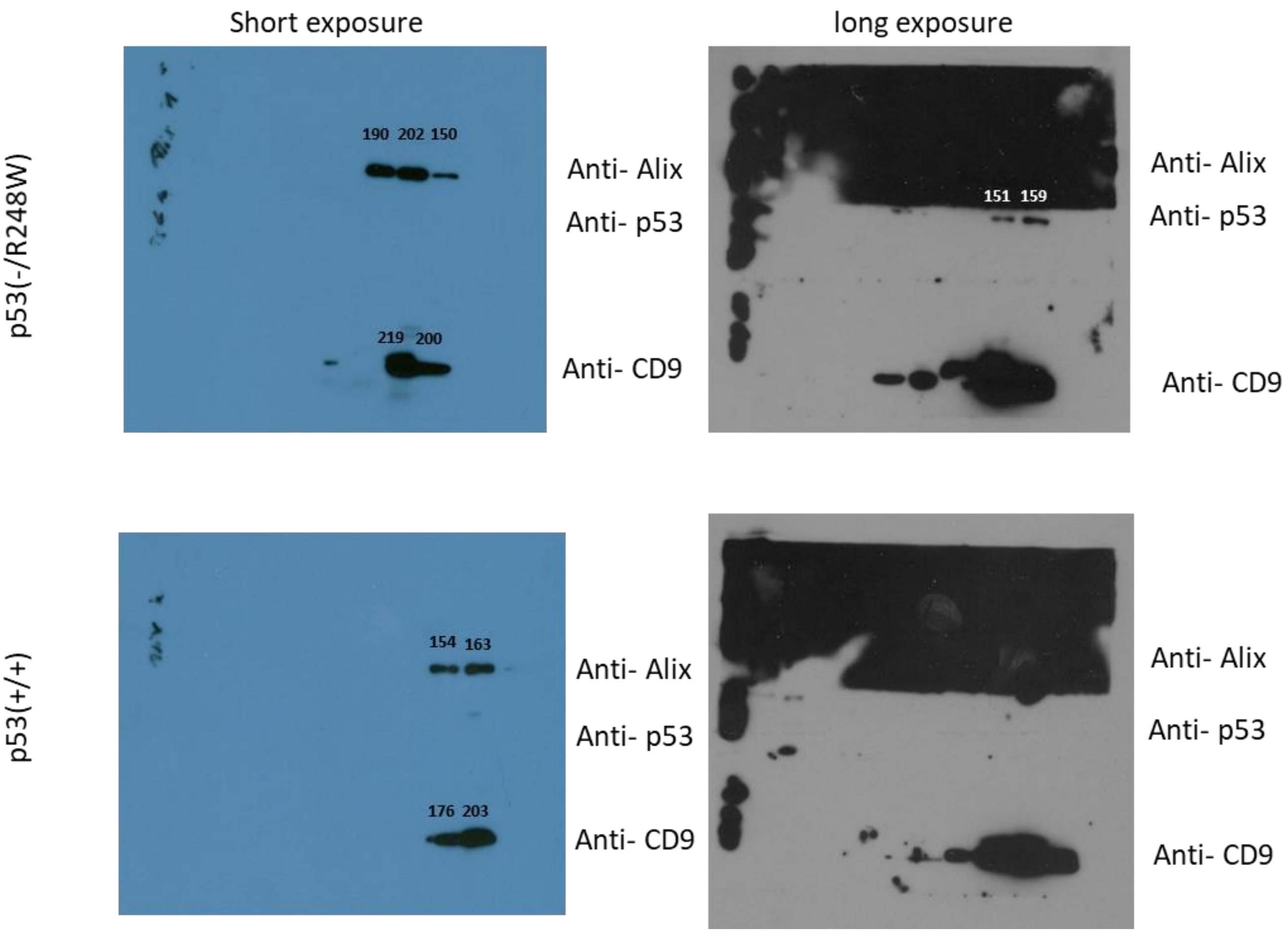

Figure S4. The whole Western Blot figure of Figure 2C

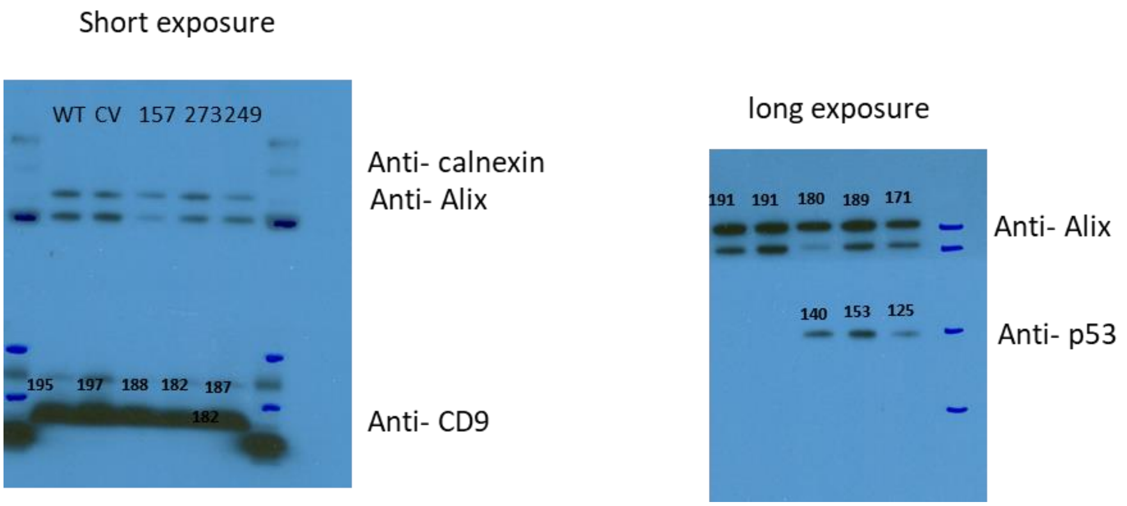

Figure S5. The whole Western Blot figure of Figure 2D

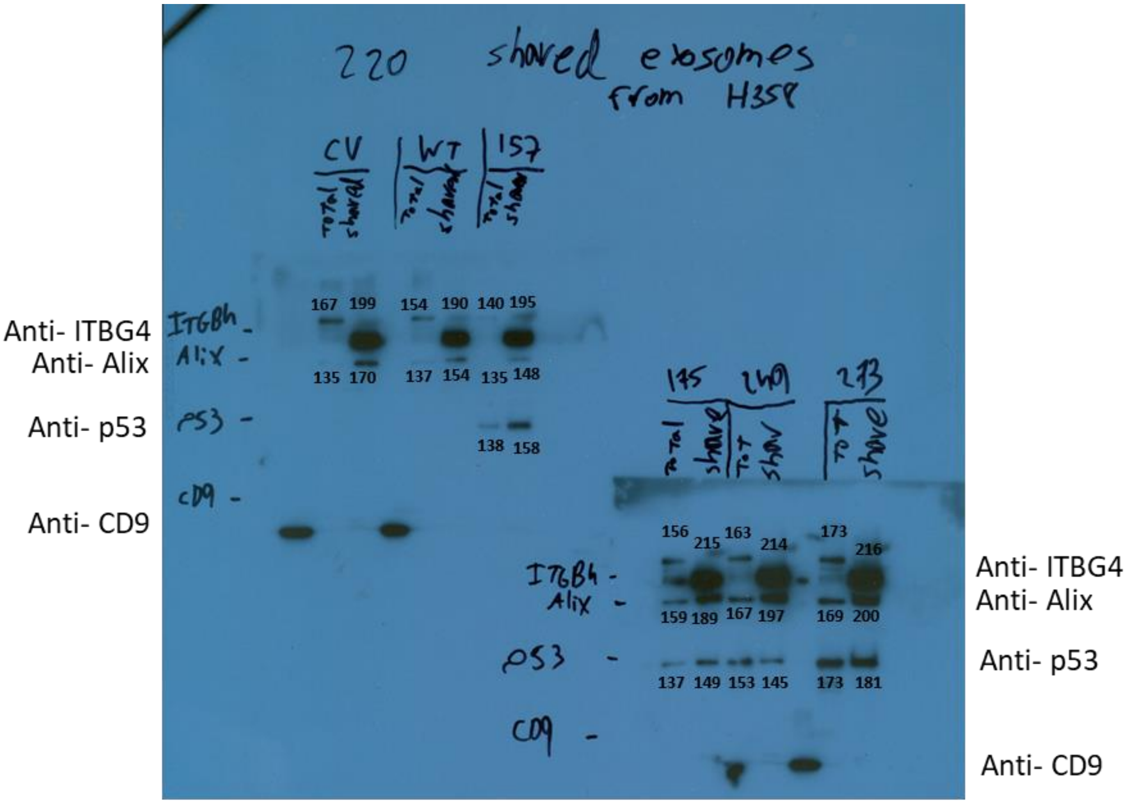

Figure S6. The whole Western Blot figure of Figure 3D

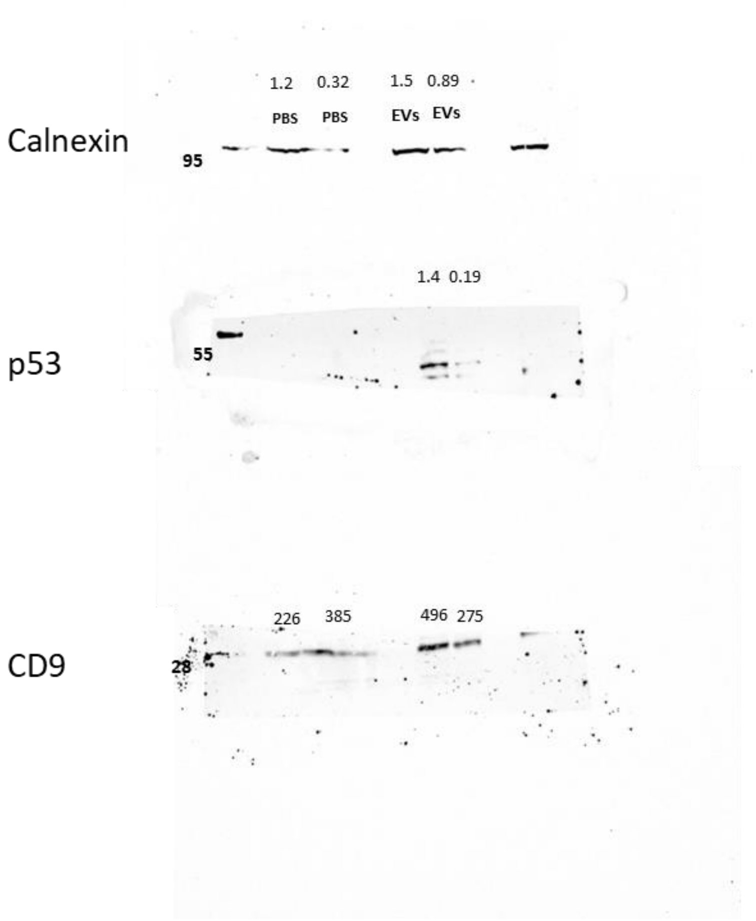

Figure S7: The whole Western Blot figure of Figure 5A

+/+ +/- +/172

α_172 (15mins)

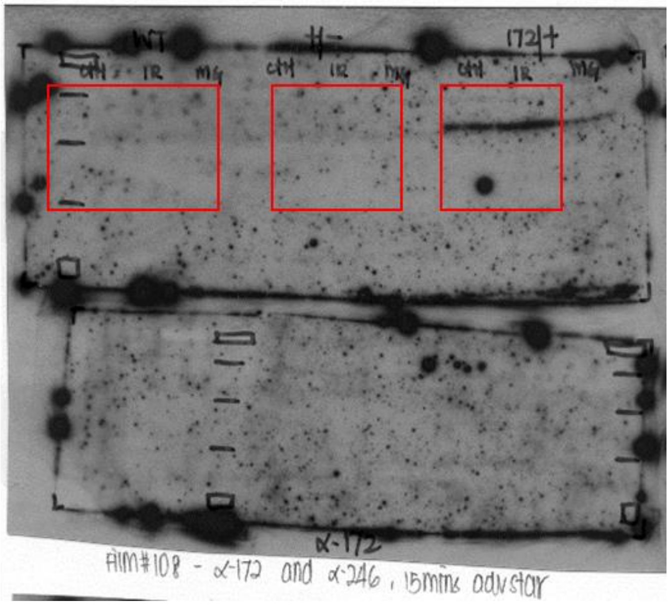

+/+ +/- +/172

α_1c12 (30sec)

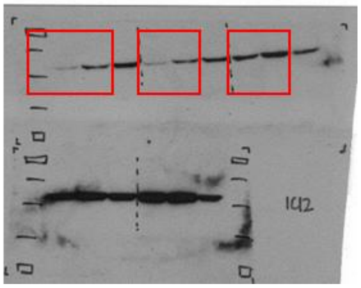

+/+ +/- +/172

α_actin (1min)

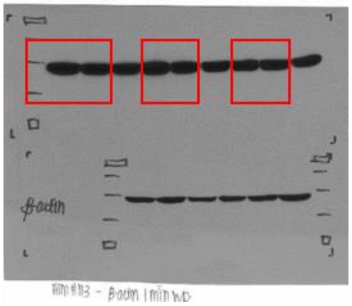

Supplement: Supplementary file 1 [file cancers-13-02985-s001.zip › cancers-1226775-SI.pdf]
